# Autonomic modulation of neuroplasticity in spinal cord injury rehabilitation: insights from a narrative review

**DOI:** 10.3389/fneur.2026.1786173

**Published:** 2026-04-09

**Authors:** Roberto Di Palma, Luigi Falco, Armando Coccia, Federica Amitrano, Giovanni D'Addio, Maria Teresa La Rovere

**Affiliations:** 1 Movement Analysis and Robotics Laboratory, Istituti Clinici Scientifici Maugeri IRCCS, Telese Terme, BN, Italy; 2 Department of Cardiology, Istituti Clinici Scientifici Maugeri IRCCS, Montescano, PV, Italy

**Keywords:** spinal cord injury, autonomic nervous system, neuroplasticity, heart rate variability, vagus nerve stimulation, rehabilitation

## Abstract

Spinal Cord Injury (SCI) causes persistent autonomic dysregulation, which is not merely a clinical epiphenomenon, but a mechanistic condition shaping the neurochemical, neurovascular, and immuno-endocrine milieu in which plasticity unfolds. Yet, the modulatory role of the Autonomic Nervous System (ANS) in post-injury circuit reorganization remains insufficiently integrated in rehabilitation paradigms. This narrative review synthesizes emerging evidence (2020–2025) describing how sympatho–vagal dynamics constrain or enable adaptive plasticity following SCI, and surveys therapeutic strategies that intentionally leverage autonomic modulation to amplify recovery. Mechanistically, autonomic tone influences neuronal excitability, perfusion, neurotrophin signaling (notably Brain-Derived Neurotrophic Factor (BDNF)/Tropomyosin receptor kinase B (TrkB)), and microglia-dependent inflammatory states. Recurrent sympathetic surges during Autonomic Dysreflexia (AD) bias networks toward maladaptive phenotypes, whereas enhanced vagal flexibility promotes neurotrophin availability, homeostatic excitability, and synaptic strengthening. Interventions including Vagus Nerve Stimulation (VNS), paired with task-specific training, respiratory-based protocols, Heart Rate Variability (HRV) biofeedback, and individualized aerobic exercise demonstrate promising autonomic and functional effects. Altogether, these observations support the view that autonomic modulation is a mechanistic boundary condition for post-injury plasticity, rather than a secondary consequence of SCI. Future work requires rigorously powered, multimodal trials integrating autonomic biomarkers—especially HRV—with neurophysiological endpoints to refine dose-specific protocols and accelerate translation into precision-based rehabilitation.

## 1 Introduction

SCI represents a pressing global health concern, affecting an estimated 20.6 million individuals worldwide as of 2019, with approximately 0.9 million new cases each year. Over the past three decades, both the prevalence and incidence of SCI have risen markedly particularly among men and older adults primarily due to falls and road traffic accidents (1–5). The consequences of SCI are extensive and enduring, encompassing not only profound physical impairment but also significant psychological and social burden. Individuals with SCI frequently contend with chronic pain, spasticity, sexual dysfunction, and a high burden of comorbid conditions, with more than 95% reporting at least one secondary health problem and an average of seven concurrent conditions per person (6, 7). Beyond the medical dimension, SCI frequently results in severe disability, diminished independence, and challenges in social reintegration, employment, and interpersonal relationships, often accompanied by psychiatric sequelae such as depression, anxiety, and increased suicidality (6). A key determinant of post-injury complications is the degree of autonomic dysfunction, which varies according to lesion level and completeness, with injuries at the cervical and upper thoracic levels producing the most pronounced autonomic impairments (8, 9).

The ANS, comprising the sympathetic and vagal divisions, is integral to maintaining physiological homeostasis through regulation of cardiovascular, respiratory, and visceral functions. Following SCI, the supraspinal regulation of ANS is disrupted especially affecting the sympathetic pathways arising from spinal segments T1–L2. This disruption results in diminished sympathetic output and unopposed vagal activity, leading to a cascade of physiological dysregulations, including cardiac arrhythmias, orthostatic hypotension, AD, bronchoconstriction, excessive respiratory secretions, and dysfunctions in bowel, bladder, and sexual systems (10). These disturbances not only compromise acute physiological stability but also contribute to chronic complications such as accelerated cardiovascular pathology, impaired cerebral perfusion, sleep disturbances, and cognitive decline, all of which substantially limit rehabilitation potential and daily functioning (9–12). Recent evidence demonstrates that chronic autonomic imbalance following SCI is linked to reduced left ventricular contractility and structural myocardial alterations, consistent with a primary cardiac inotropic impairment driven by loss of descending sympathetic control (13).

Particularly, cardiovascular instability and AD constitute serious clinical risks, with direct implications for quality of life and increased mortality in this population (10–12). Early identification and management of autonomic dysfunction are, therefore, essential to improving long-term rehabilitation outcomes and patient wellbeing (8, 9, 14). The connection between ANS, neuroplasticity, and recovery after SCI is rooted in the nervous system's capacity to adapt and reorganize following trauma. Through the regulation of cerebral blood flow, neurotrophic factor distribution, and metabolic support, ANS creates conditions conducive to adaptive neuroplasticity while potentially mitigating maladaptive responses (15). In this context, autonomic dysfunction may hinder recovery by disrupting these regulatory mechanisms, whereas interventions that restore autonomic balance could enhance the efficacy of rehabilitation efforts (16–18). Advances in understanding the cellular and molecular mechanisms such as the role of neurotrophic factors, interneuron subtype-specific plasticity, and remodeling of spinal circuits are informing new therapeutic strategies that target both autonomic and somatic systems (17, 19–21). Integrative approaches that combine neuromodulation, rehabilitation, and regenerative therapies are increasingly effective in enhancing neuroplasticity and functional recovery after SCI (17, 20, 22, 23).

Unlike systematic reviews focused narrowly on isolated interventions, this narrative review offers a multidimensional perspective on how autonomic modulation influences recovery trajectories after SCI. Despite growing recognition of the interplay between autonomic regulation and Central Nervous System (CNS) recovery, this area remains underexplored in the context of SCI rehabilitation. By examining physiological mechanisms, emerging therapeutic strategies, and clinical implications, we seek to highlight how autonomic dysfunction may hinder recovery and conversely, how restoring autonomic balance could enhance neuroplastic potential. This non-systematic approach allows for a broader, integrative perspective across disciplines and sources, offering a conceptual framework to inform future research and clinical practice. We focus both on the deleterious effects of autonomic dysfunction and on the therapeutic potential of autonomic modulation, including VNS and neuromodulatory interventions, as drivers of neuroplasticity and recovery.

## 2 Methodology

This narrative review adopts a scoping-like approach, designed to map current knowledge and conceptual trends concerning the role of ANS activity in modulating neuroplasticity and influencing functional recovery following SCI. While not conducted according to the strict protocols of systematic reviews (e.g., PRISMA), this method allows for a broader interdisciplinary perspective that integrates clinical, experimental, and translational evidence. The search strategy was conceptually driven and iteratively refined to ensure comprehensive coverage of autonomic subdivisions and neuromodulatory mechanisms.

Literature searches were performed in PubMed, Scopus, and Web of Science, using combinations of keywords and controlled vocabulary (MeSH terms where applicable), including: “spinal cord injury,” “autonomic nervous system,” “sympathetic,” “parasympathetic,” “neuroplasticity,” “autonomic dysfunction,” “vagus nerve stimulation,” “neuromodulation,” “rehabilitation,” and “functional recovery.”

**Figure 1:**
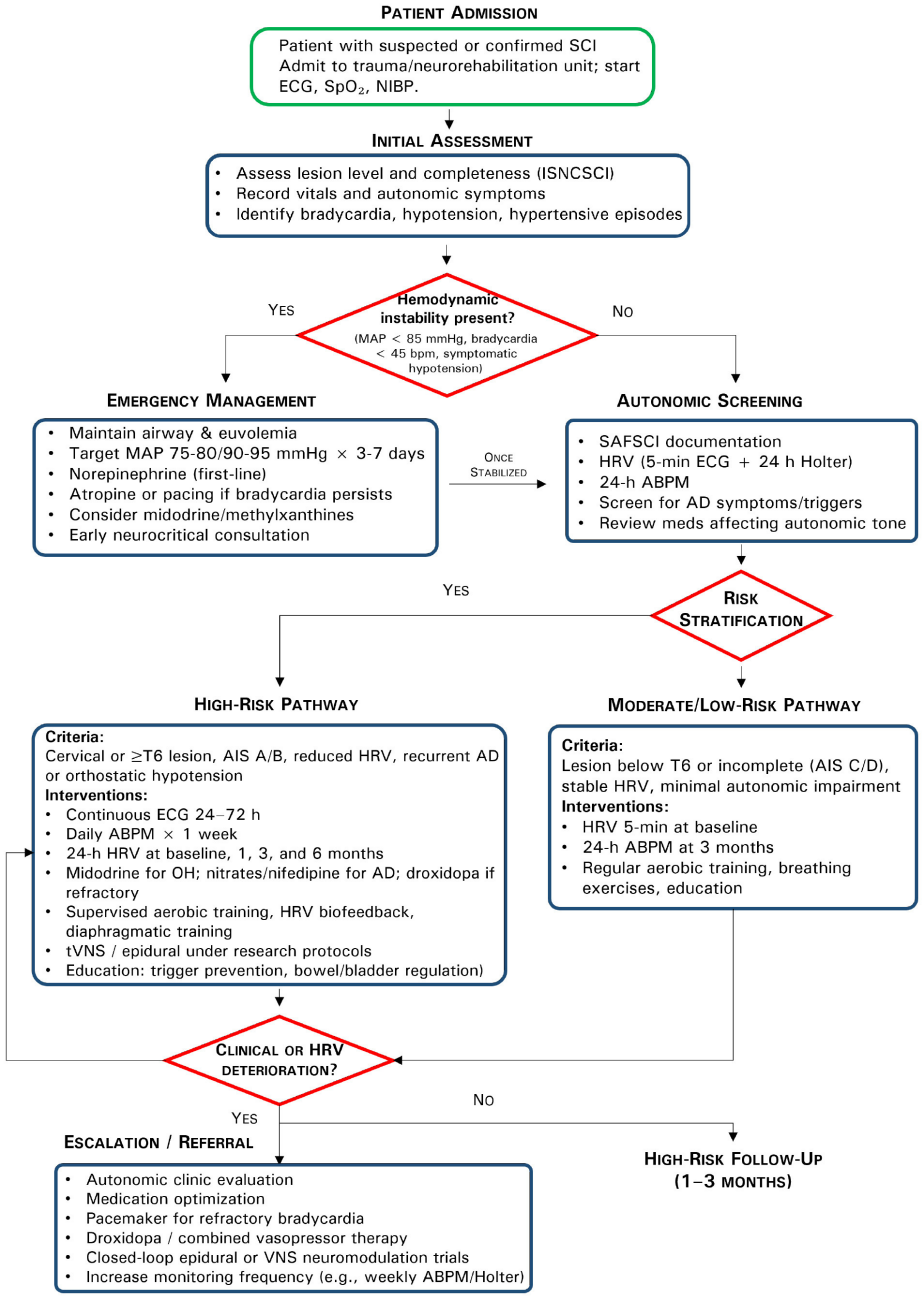
Clinical algorithm for screening, risk stratification, targeted interventions, and monitoring of autonomic dysfunction after SCI.

Boolean operators were applied to optimize specificity and sensitivity. We prioritized studies published between 2020 and 2025 to ensure the review reflects current advances and trends in the field. However, seminal or highly cited earlier works were retained when they contributed essential theoretical or mechanistic insights. Inclusion criteria were: (i) studies involving human subjects or animal models with direct relevance to SCI or autonomic modulation; (ii) focus on mechanisms of neuroplasticity, neuromodulation, or rehabilitation; (iii) publication in peer-reviewed journals. Articles were excluded if they lacked original data, focused exclusively on pharmacological treatment without behavioral/rehabilitative implications, or were not published in peer-reviewed sources. The overall clinical framework for screening, risk stratification, and management of autonomic dysfunction after SCI is summarized in Figure 1.

Article selection was based on relevance to the research question, with a focus on mechanistic clarity, translational applicability, and clinical significance. A snowballing strategy was also used to identify additional relevant sources from the references of included papers.

Given the narrative nature of this review, no formal risk of bias assessment or quantitative data synthesis was conducted. Instead, the emphasis is on conceptual synthesis, aiming to identify emerging patterns, therapeutic hypotheses, and potential gaps in current knowledge regarding the intersection between autonomic modulation and neuroplasticity in SCI rehabilitation.

## 3 Functional anatomy of the autonomic nervous system

ANS is a complex, involuntary control network responsible for maintaining internal homeostasis, including cardiovascular, gastrointestinal, thermoregulatory, metabolic, and endocrine functions. It comprises three main subdivisions: the sympathetic, parasympathetic, and Enteric Nervous System (ENS)s, each with distinct anatomical features, neurotransmitters, and functional targets (24, 25).

The Sympathetic Nervous System (SNS) originates in the thoracolumbar segments of the spinal cord (T1–L3), where preganglionic neurons located in the intermediolateral cell column project to paravertebral or pre-vertebral ganglia. These ganglia function as relay centers, where Acetylcholine (ACh) activates nicotinic receptors on postganglionic neurons, which in turn release Norepinephrine (NE) to act on adrenergic receptors at the effector organs (24–26). This system is responsible for the generalized “fight-or-flight” response, with widespread effects including increased heart rate, vasoconstriction, bronchodilation, and inhibition of gastrointestinal motility (25).

The Parasympathetic Nervous System (PNS) arises from the craniosacral regions—specifically, cranial nerves III, VII, IX, and X from the brainstem, and the sacral spinal cord segments (S2–S4). Preganglionic fibers travel long distances to ganglia located near or within the target organs, where they synapse with postganglionic neurons using ACh, which also acts at muscarinic receptors (24–26). PNS is responsible for "rest-and-digest" activities, such as digestion, urination, salivation, and sexual arousal. Its actions are generally more localized and organ-specific compared to the diffuse responses of SNS (25).

However, recent research has challenged the classic view, suggesting that the sacral autonomic outflow may share more characteristics with the sympathetic system than previously thought, potentially redefining the boundaries between sympathetic and parasympathetic divisions in the sacral region (27, 28). Despite this debate, the consensus remains that sympathetic fibers exit the spinal cord between T1 and L2, while parasympathetic fibers are craniosacral, with cranial outflow via brainstem nuclei and sacral outflow via S2–S4 (29, 30). Disruption of these pathways, such as through SCI, can lead to significant autonomic dysfunction, affecting cardiovascular, gastrointestinal, and genitourinary systems (29).

The sympathetic pathways are especially vulnerable to disruption in SCI, leading to significant autonomic dysfunction depending on the level of injury (10, 31–33). Parasympathetic pathways, particularly those from the sacral cord, can also be affected, resulting in issues with bladder, bowel, and sexual function (31). Overall, the thoracolumbar spinal cord is the main origin for sympathetic outflow, while the sacral spinal cord is key for parasympathetic outflow to pelvic organs, with both systems relying on complex spinal and supraspinal regulation (30, 31, 33).

Often described as the “second brain,” the ENS consists of an intrinsic network of neurons embedded within the gastrointestinal tract. It functions independently, though it receives modulatory input from both sympathetic and parasympathetic divisions. The ENS regulates intestinal motility, secretion, and local blood flow through coordinated interactions between the myenteric and submucosal plexuses, and it integrates signals from vagal and spinal afferents (25, 34, 35).

### 3.1 Bidirectional interaction with the central nervous system

ANS is tightly regulated by supraspinal centers, especially the hypothalamus and brainstem nuclei (e.g., the nucleus tractus solitarius, rostral ventrolateral medulla). These centers receive sensory inputs from baroreceptors, chemoreceptors, and visceral afferents, and generate appropriate efferent autonomic responses. The hypothalamus plays a central role in integrating autonomic, endocrine, and behavioral responses to stress, fluid balance, thermoregulation, and circadian rhythms. Moreover, higher cortical regions, including the insular and midcingulate cortex, modulate autonomic output through emotional and cognitive processing, which explains phenomena like stress-induced tachycardia and vasovagal syncope (36, 37). This bidirectional communication enables the CNS to adjust autonomic tone based on both internal and external stimuli, while autonomic signals can influence brain function via humoral and neural routes, particularly through the vagus nerve and Hypothalamic Pituitary Adrenal (HPA) axis (25, 37). Chronic activation of these systems can lead to pathophysiological conditions such as hypertension, immune dysregulation, and mood disorders (27).

## 4 Autonomic modulation of neuroplasticity: mechanisms and evidence

The ANS, through its sympathetic and parasympathetic branches, exerts a significant modulatory influence on neuroplasticity by acting at multiple levels of neuronal regulation, including excitability, neurovascular coupling, neurotrophin signaling, stress hormone balance, and cortical reorganization (37–41). At the level of neuronal excitability, ANS modulates the electrical responsiveness of central and peripheral neurons via neurotransmitters such as NE (sympathetic) and ACh (parasympathetic), as well as co-released neuropeptides (41). These modulators affect membrane potentials and ion channel dynamics, thereby adjusting the synaptic activation threshold and the probability of action potential firing.

In the CNS, sympathetic noradrenergic modulation is highly receptor- and state-dependent. Acute NE release acting at β-adrenergic receptors (β-ARs) in hippocampal CA1 and ventral subiculum facilitates N-Methyl-D-Aspartic acid (NMDA)-dependent Long-Term Potentiation (LTP) and lowers its induction threshold, particularly under acute stress conditions (42). Activation of β-ARs can also reshape complex-spike–dependent plasticity by enhancing potentiation at active synapses while imposing heterosynaptic competition, thereby preventing runaway excitation (43). In contrast, activation of α1-adrenergic receptors (α1-ARs) can induce Long-Term Depression (LTD) at CA3–CA1 synapses and in visual cortex via postsynaptic modulation of AMPA/NMDA receptors and PLC–IP_3_ signaling (44). Chronic stress alters this balance: prolonged glucocorticoid exposure reduces hippocampal BDNF and NeuroTrophin-3 (NT-3) expression (45–47), disrupts α1-ARs–dependent LTD (48), and impairs LTP and synaptic homeostasis (49–51). Sympathetic signaling appears to promote adaptive plasticity when transient, whereas sustained activation favors maladaptive remodeling.

At the same time, parasympathetic activity, particularly through vagal afferents, modulates central plasticity both directly and indirectly. Although no experimental paradigm has directly compared Locus Coeruleus (LC) activation with vagal stimulation within the same hippocampal preparation, converging evidence indicates that VNS enhances hippocampal LTP, increases CA1 firing, and elevates BDNF expression in hippocampus and cortex (40, 52). Importantly, VNS increases central NE release and recruits LC β-AR signaling; β-blockade prevents VNS-induced enhancement of perforant path–CA3 transmission (52, 53), demonstrating convergence with noradrenergic plasticity pathways. In addition, VNS activates cortical cholinergic and noradrenergic projections and induces structured increases in network excitability rather than global inhibition (54). These findings suggest that parasympathetic engagement promotes experience-dependent plasticity through neuromodulatory coordination rather than simple suppression of activity.

Cerebral and spinal blood flow are also dynamically regulated by autonomic tone. Sympathetic activity induces vasoconstriction and stabilizes perfusion under systemic stress, whereas parasympathetic input, particularly through cranial autonomic outflow, facilitates vasodilation in metabolically active regions (36, 39). Although autonomic inputs are not the primary drivers of task-evoked neurovascular coupling (55, 56), disrupted autonomic regulation after SCI has been associated with impaired cerebral autoregulation and increased vascular risk (57, 58), potentially limiting the metabolic support required for adaptive plasticity.

Maladaptive plasticity in autonomic circuits—exacerbated by microglial activation—can amplify dysautonomia and delay recovery (15, 56). Chronic sympathetic overactivity has been associated with early microglial activation and neuroinflammation in autonomic control regions (59, 60), whereas vagal stimulation activates the cholinergic anti-inflammatory pathway via α7 nicotinic receptors, suppressing Tumor Necrosis Factor-α (TNF-α) and Nuclear Factor kappa-light-chain-enhancer of activated B cells (NF-kB)–dependent signaling (61–63). In rodent models of endotoxemia and chronic stress, VNS reduces hippocampal microglial activation and restores cognitive or affective performance, linking autonomic modulation, neuroinflammation, and functional outcomes.

The interaction between autonomic signaling and neurotrophin regulation emerges as a key integrative node linking synaptic plasticity, inflammation, and structural remodeling.

ANS also directly influences neurotrophin expression and signaling in both central and peripheral compartments. Both sympathetic and parasympathetic activity modulate levels of BDNF, Nerve Growth Factor (NGF), and NT-3, which are critical for synaptic plasticity, axonal regeneration, and circuit reorganization. In the CNS, chronic stress reduces hippocampal BDNF and NT-3 expression (45, 46), and lower hippocampal BDNF is strongly associated with depressive-like and stress-susceptible phenotypes (64, 65). Vagal nerve activity in rats supports basal BDNF expression (40), and VNS induces phosphorylation/activation of its TrkB receptor (66), while restoring NGF expression under stress conditions (67–69).

Beyond BDNF–TrkB pathways, vagal modulation appears to critically influence NGF-dependent mechanisms: in spinal cord injury models, VNS reduces neuroinflammation by shifting microglial polarization from a pro-inflammatory (M1) to a reparative (M2) phenotype, promoting functional recovery (67). Given the role of NF-kB in regulating neurotrophin transcription under inflammatory conditions, vagal engagement may stabilize NGF expression by restoring neuroimmune balance within injured circuits. At the peripheral level, autonomic stress responses directly modulate NGF–Tropomyosin receptor kinase A (TrkA) signaling. Chronic stress increases arterial NGF levels and TrkA receptor expression, driving Vascular Endothelial Growth Factor (VEGF)-dependent vascular remodeling; these effects are attenuated by NGF neutralization (68), demonstrating that NGF functions as an effector of stress-induced neurovascular adaptation. More directly, auricular VNS suppresses central NGF/TrkA/Phospholipase C-γ (PLC-γ) signaling in models of functional dyspepsia with gastric hypersensitivity (52). Vagal stimulation reduces NGF expression, TrkA activation, and downstream signaling, thereby attenuating neuronal sensitization, while pharmacological Tropomyosin Receptor Kinase (Trk) inhibition produces comparable effects. Together, these findings indicate that the autonomic nervous system exerts bidirectional control over NGF signaling. Sympathetic activation may enhance NGF-driven remodeling, whereas vagal engagement—via α7nAChR-dependent modulation of inflammatory signaling—appears to recalibrate NGF–Trk pathways toward a regulated and adaptive state.

In several models—including hypoxia, Parkinson's disease, and chronic stress—VNS-induced increases in BDNF and/or NGF correlate with improvements in memory, motor function, or affective behavior (60, 70, 71). Pharmacological blockade of TrkB signaling attenuates specific neuroprotective or antidepressant-like effects (70), indicating partial causal involvement of BDNF–TrkB pathways.

In the PNS and visceral systems, NGF displays context-dependent effects. Elevated peripheral NGF contributes to visceral hypersensitivity, and anti-NGF strategies normalize nociceptive thresholds in models of colonic and gastric hypersensitivity (72–74). Conversely, NGF can promote anti-inflammatory and pro-repair phenotypes in both peripheral nerve injury and selected CNS contexts (75, 76). Thus, restoration of NGF toward physiological levels may support repair and autonomic stability, whereas excessive NGF in peripheral tissues may be pro-nociceptive. Although direct causal evidence linking vagal modulation to NGF-mediated anti-inflammatory effects remains limited, converging data support NGF as a plausible integrator of autonomic, immune, and plasticity-related processes.

While stress-related effects have been discussed at the synaptic and neurotrophin levels, it is important to explicitly consider the integrated autonomic–endocrine axis.

Chronic stress, via sustained activation of the HPA axis and autonomic efferents, disrupts synaptic homeostasis by elevating cortisol and catecholamine levels. Prolonged exposure to glucocorticoids impairs LTP, enhances GABAergic tone, and induces microglia-mediated synaptic pruning, contributing to cognitive decline and vulnerability to affective disorders (49–51). Whereas acute stress responses may transiently enhance certain forms of plasticity, chronic autonomic-endocrine feedback loops are more often associated with neurodegenerative rather than adaptive outcomes (77, 78).

Among the most promising strategies for therapeutically harnessing autonomic modulation is VNS, which has been shown to facilitate cortical plasticity by modulating noradrenergic and serotonergic tone, increasing BDNF availability, and enhancing motor map reorganization in both preclinical and clinical contexts (41). Pairing VNS with rehabilitative training amplifies experience-dependent synaptic strengthening and improves functional recovery after peripheral nerve injury and stroke (79–81). In the context of SCI, neuromodulatory strategies such as VNS are being explored for their potential to guide spinal and cortical circuit reorganization in a targeted, activity-dependent manner (81–83), potentially combining β-adrenergic–mediated facilitation of synaptic plasticity with vagal-driven neuroimmune stabilization.

Collectively, these findings support a model in which autonomic tone functions as a dynamic regulatory axis. It coordinates synaptic, vascular, immune, and trophic plasticity across central and peripheral compartments: sympathetic activation, particularly via β-AR signaling, may enhance plasticity when transient and task-coupled, whereas parasympathetic/vagal engagement appears especially suited to restoring homeostatic balance, limiting neuroinflammation, and supporting neurotrophin-dependent repair. Therapeutic modulation of this axis may therefore represent a systems-level strategy for optimizing adaptive remodeling following CNS injuries.

## 5 Autonomic nervous system and spinal cord injury

Following SCI, disruption of descending autonomic pathways impairs sympathetic vascular control, resulting in hemodynamic instability, orthostatic hypotension, and AD—thereby compromising cerebral and spinal perfusion (9, 26, 56). Consequently, oxygen and nutrient delivery is impaired, limiting substrates essential for neuroplastic mechanisms. The severity of autonomic dysfunction depends on the level and extent of damage to the spinal/central component of ANS. High-level SCI (T6 or above) abruptly interrupts supraspinal inhibition of sympathetic pre-ganglionic neurons, setting the stage for the development of classical AD triad—noxious or innocuous stimulus below the lesion → explosive spinal sympathetic reflex → failure of supraspinal modulation—that exemplifies maladaptive plasticity of spinal autonomic circuits (58, 84). In the acute phase, this disruption causes profound hemodynamic changes due to loss of sympathetic outflow, resulting in neurogenic shock characterized by severe hypotension and bradycardia, secondary to unopposed parasympathetic (vagal) activity (14, 85–88). Neurogenic shock typically develops within hours, peaking at day 4, and generally lasts 1–3 weeks, although severe cases may persist up to 5 weeks (86, 89, 90). Bradycardia and hypotension are most pronounced during the first week but may persist beyond this period in complete cervical lesions (14, 85). Recent guidelines suggest that mean arterial pressure (MAP) should be augmented to a lower limit of 75–80 mmHg and not actively augmented beyond an upper limit of 90–95 mmHg for a duration of 3–7 days post-injury. This recommendation is based on very low-quality evidence, with no specific preference for the choice of vasopressor (87, 88). Vasopressors remain the mainstay, with NE increasingly preferred over dopamine or phenylephrine due to superior spinal cord perfusion and fewer cardiac complications, although agent selection should be individualized (88, 91, 92). Midodrine and methylxanthines (theophylline, aminophylline) are considered for persistent hypotension and bradycardia, while enteral albuterol and droxidopa are emerging options (93–95). In the chronically injured cord, sprouting of calcitonin gene related peptide-positive afferents, recruitment of excitatory propriospinal interneurons and heightened α1-AR sensitivity transform routine bladder distension or cutaneous pressure into paroxysmal hypertension, reflex bradycardia and arrhythmogenic risk severe enough to precipitate stroke or sudden death if untreated (12, 96). These episodic surges coexist with a depressed baseline sympathetic tone that drives orthostatic hypotension, blunted Blood Pressure Variability (BPV), dyslipidemia and systemic low-grade inflammation, thereby compounding cardiovascular and metabolic morbidity (97). Parasympathetic (vagal) pathways remain anatomically intact above the lesion, yet the loss of sympathetic counterbalance often leaves unopposed vagal dominance—manifesting as resting bradycardia and impaired chronotropic reserve (98). HRV assessment is widely used as non-invasive method to evaluate sympathetic and parasympathetic cardiac modulation in the heart of patients with SCI. HRV refers to the variation in time intervals between heartbeats, which can be detected using a 24-hour ElectroCardioGram (ECG) recording or a short-term resting supine ECG (typically five minutes).

Among the numerous HRV-derived indices, the literature consistently highlights Standard Deviation of Normal-to-Normal intervals (SDNN), Root Mean Square of Successive Differences (RMSSD), Low Frequency (LF) power, High Frequency (HF) power, and the LF/HF ratio as the most frequently reported parameters (99–109). In particular, RMSSD and HF power are widely recognized as the standard indices of parasympathetic (vagal) cardiac modulation, while standard deviation of SDNN reflects global HRV. In contrast, LF has been the subject of much debate in the literature. While some studies have concluded that LF power reflects a combination of sympathetic and parasympathetic influences, other data indicate that LF oscillations in heart rate mainly represent vagal transmission of corresponding Blood Pressure (BP) oscillations via the baroreflex (110). The LF/HF ratio has long been used to describe the so-called “sympathovagal balance” (111). However, this concept has been heavily criticized on the grounds that it lacks a precise definition and sound physiological basis (112, 113). Current clinical standards such as the International Standards for Neurological Classification (ISNC) of SCI and the International Standards to document remaining Autonomic Function (ISAF) after SCI are not sensitive enough to detect autonomic function. It has been recently suggested that bedside HRV assessment could help to identify different degrees of autonomic impairment highlighting the need for more accessible and accurate HRV-based monitoring in rehabilitation settings (114). Rehabilitation strategies that also have the potential to improve HRV may support the use of HRV as a biomarker and therapeutic target in SCI recovery. In other words, since HRV reflects autonomic modulation, which influences neurotrophin availability, cerebral and spinal perfusion, and inflammatory responses, it can be considered an indirect yet clinically accessible indicator of the neuroplastic environment. While studies in SCI populations have primarily focused on documenting autonomic–cardiac uncoupling and the utility of HRV measures for assessing dysfunction (114, 115), growing evidence from stroke, traumatic brain injury, and preclinical SCI models indicates that interventions such as aerobic exercise, neuromodulation, and VNS that can modulate HRV are associated with improvements in motor function and daily living activities (80, 116–120). Importantly, autonomic imbalance following SCI adversely affects the neural substrate for plasticity. Recurrent surges in sympathetic activity during AD expose the circulation to excessive levels of NE and glucocorticoids. This may bias lumbar locomotor networks toward long-term depression, suppresses BDNF–TrkB signaling, and promotes microglia-mediated synaptic pruning. Conversely, enhanced vagal release of ACh and anti-inflammatory cytokines, restores neurotrophin availability, and re-establishes an environment conducive to adaptive circuit remodeling (121, 122). Thus, the autonomic milieu after SCI is both a determinant of systemic morbidity and a gatekeeper of neuroplastic potential.

Functional recovery after SCI depends on activity-dependent plasticity within spared descending pathways, propriospinal circuits, and segmental motor networks (79, 80, 82). These processes require adequate neurotrophin signaling—particularly BDNF–TrkB and NGF–Trk pathways—to support axonal sprouting, synaptic stabilization, dendritic remodeling, and adaptive remodeling (40, 45, 70). Experimental evidence indicates that reduced BDNF availability impairs corticospinal tract plasticity and limits locomotor recovery, whereas enhancement of BDNF signaling facilitates synaptic strengthening and motor map reorganization (70, 71). Similarly, tightly regulated NGF signaling contributes to neuronal survival and neuroimmune stabilization within injured tissue (67, 75). Autonomic dysregulation following SCI—characterized by sympathetic hyper-reflexia, recurrent catecholamine surges, and impaired vagal modulation—may therefore constrain recovery not only through hemodynamic instability but also by suppressing neurotrophin expression and promoting microglia-mediated synaptic loss (45, 49, 60). Conversely, strategies that restore autonomic balance—such as aerobic training, neuromodulation, or VNS—have been associated with increased BDNF availability and attenuation of neuroinflammatory signaling (40, 41, 70). By recalibrating the neuroimmune–neurotrophic milieu, autonomic-targeted interventions may enhance the permissiveness of spinal and supraspinal circuits to activity-based rehabilitation. This may facilitate the translation of molecular modulation into functional gains (79, 83). Consequently, mitigating sympathetic hyper-reflexia and reinforcing vagal flexibility may be necessary to maximize the restorative potential of activity-based and neuromodulatory rehabilitation.

The disruption of the autonomic cardiovascular control results in unstable BP, with exaggerated beat-to-beat and longer-term variability, which increases the risk of cardiovascular disease. In individuals with chronic SCI, 24-h ambulatory monitoring reveals significantly greater instability of both systolic and diastolic BP compared with uninjured controls (123).

Assessing BPV provides an additional measure of autonomic cardiovascular regulation following SCI. During the acute phase, power in the LF component of BPV has been suggested as a quantitative measure of autonomic injury, with sustained reductions indicating autonomic dysfunction over several months post-injury (124). Furthermore, increased BPV in the first 24 h after acute cervical SCI has been linked to poorer functional outcomes after six months, suggesting that stabilizing BP fluctuations early on may be important for recovery (67).

## 6 Clinical and therapeutic implications

### 6.1 Vagus nerve stimulation

VNS may modulate neuroplasticity after SCI primarily by influencing neurotransmitter release (notably NE and ACh), reducing neuroinflammation, and promoting beneficial microglial polarization, which together enhance synaptic connectivity and functional recovery (67, 125–127). VNS has been shown in animal models to improve motor function, reduce scar formation, and protect the blood spinal cord barrier by inhibiting pro inflammatory cytokines and preventing endothelial necroptosis (67, 125). Closed loop VNS, especially when paired with successful motor training, maximizes recovery by timing stimulation to reinforce adaptive neural circuits, with benefits persisting long after therapy ends (126, 128). Both invasive and transcutaneous (noninvasive) VNS are under investigation; noninvasive approaches are highlighted for their safety and ease of integration into clinical practice, though direct comparative data on efficacy and tolerability in SCI patients remain limited (129, 130). Importantly, preclinical work from the Krassioukov laboratory demonstrated that chronic cervical VNS can be safely delivered in a rodent model of SCI without exacerbating autonomic instability or inducing adverse cardiovascular events, supporting the feasibility of VNS application in the context of impaired supraspinal autonomic control (131). Optimal stimulation parameters in preclinical studies often involve brief, precisely timed bursts delivered during or immediately after successful motor tasks, but the best frequency, amplitude, and duration for humans are still being refined (126, 128, 129). Clinical evidence in SCI is emerging, with animal and early human studies reporting improvements in motor recovery, quality of life, and autonomic function (including HRV), though large scale trials are still needed (127, 129, 130, 132). VNS may also alleviate non-motor complications such as pain and cardiovascular dysfunction, further supporting its therapeutic potential in SCI (130, 132). Overall, VNS represents a promising adjunct to rehabilitation, but further research is required to optimize protocols and confirm long term safety and efficacy in diverse SCI populations (127, 129, 130).

### 6.2 Acute intermittent hypoxia

Respiratory-based interventions represent a promising strategy for modulating both autonomic regulation and spinal neuroplasticity following SCI. Because respiratory control is closely integrated with autonomic and spinal motor networks, targeted manipulation of respiratory stimuli can influence neural excitability and promote plasticity within residual pathways (21, 133, 134).

Among these approaches, Acute Intermittent Hypoxia (AIH) represents a mechanistically distinct and well-characterized stimulus for inducing neuroplasticity. Unlike volitional respiratory training, AIH consists of brief, repeated hypoxic exposures interspersed with normoxia that trigger serotonin-dependent signaling cascades within the spinal cord, particularly via 5-HT_2_ receptor activation, leading to BDNF synthesis and TrkB receptor activation in phrenic motor networks. These mechanisms underlie phrenic Long-Term Facilitation (pLTF), a sustained enhancement of respiratory motor output extensively characterized in preclinical models of cervical SCI (134, 135). Notably, the capacity to express AIH-induced pLTF is state-dependent; sex hormone supplementation restores respiratory neuroplasticity following C2 hemisection in rodents, highlighting endocrine modulation of BDNF-dependent pathways (135).

Beyond respiratory motor nuclei, AIH induces plasticity within descending motor pathways. In humans, a single session of AIH enhances corticospinal synaptic efficacy, increasing motor-evoked potential amplitudes without altering intracortical inhibition, consistent with strengthened corticospinal–motoneuronal transmission at the spinal level (136). In individuals with tetraplegia, AIH augments spinal plasticity and voluntary motor output, supporting its translational relevance in individuals with chronic cervical SCI (137). Importantly, AIH-induced enhancements in corticospinal excitability predict improvements in motor learning and metabolic efficiency (138), supporting the concept that AIH, while delivered through respiratory manipulation, functions as a systemic metaplastic priming stimulus capable of amplifying task-specific rehabilitation effects.

Mechanistically, AIH engages multiple interacting pathways. The canonical serotonergic pathway (“Q pathway”) is initiated by hypoxia-induced activation of brainstem raphe neurons, triggering spinal Gq protein-coupled 5-HT2 receptor signaling, protein kinase C and Extracellular signal-Regulated Kinase (ERK) Mitogen-Activated Protein (MAP) kinase activation, and BDNF synthesis, which sustains pLTF in phrenic motor neurons (139–141). In parallel, adenosine-dependent pathways (“S pathway”) are recruited particularly under severe hypoxic stress or in the context of reduced spinal oxygen tension, leading to TrkB-mediated plasticity independent of BDNF synthesis (139, 140, 142). While the serotonergic (Q) and adenosine-dependent (S) pathways underlying AIH-induced plasticity are well-characterized in phrenic motor neurons, whether these same molecular mechanisms operate similarly within autonomic circuits remains to be fully elucidated, though AIH clearly elicits sympathetic long-term facilitation post-SCI. Interactions between serotonergic and adenosinergic mechanisms through cross-talk inhibition modulate net plasticity outcomes, while chronic SCI may diminish BDNF upregulation, shifting reliance toward adenosinergic mechanisms (143). In chronic SCI, AIH-induced BDNF upregulation within phrenic motor neurons is substantially limited, indicating that plasticity in these circuits may rely more heavily on adenosine-mediated mechanisms rather than serotonin-dependent pathways (143). AIH-induced plasticity is also influenced by biological variables, including sex hormones, peripheral chemoreflex sensitivity, and the timing of the post-injury period. Over time, partial serotonergic reinnervation has been shown to restore capacity for serotonin-dependent responses (141, 142, 144).

Specifically, the hypothesis is that sex hormones may modulate autonomic plasticity through effects on serotonergic signaling and BDNF expression. In addition, heightened chemoreflex sensitivity has been shown to amplify sympathetic responses, although sympathetic Long-Term Facilitation (sLTF) may also occur independently of peripheral chemoreceptor inputs (141, 144, 145). The predominance of serotonin- or adenosine-dependent mechanisms is determined by the timing and severity of the injury, with the resultant influence on the magnitude of both motor and autonomic facilitation (140, 142).

It is important to note that the effects of AIH extend beyond motor systems to autonomic and cardiovascular networks. Preclinical studies demonstrate that AIH instigates sympathetic long-term facilitation (sLTF) in splanchnic and renal sympathetic circuits following SCI, independently of peripheral chemoreflex activation or spinal tissue hypoxia. This supports the hypothesis that there is enhanced sympathetic output following AIH post-injury (142, 145). AIH-induced sLTF has been demonstrated to mitigate SCI-associated sympathetic hypoactivity and hypotension (146). In addition to this, the condition has also been shown to produce acute enhancements in cardiac function, including left ventricular contractility, arterial pressure, and coronary perfusion. These effects are likely to be a result of potentiated sympathetic outflow. Clinical studies in individuals with chronic SCI have confirmed that mild-to-moderate AIH is generally safe, well tolerated, and capable of selectively enhancing ventilatory and heart rate responses without inducing sustained cardiovascular perturbations. However, interindividual variability is substantial (144).

Collectively, these findings lend support to the hypothesis that AIH functions as a systemic metaplastic primer, capable of enhancing synaptic and network plasticity across motor and autonomic domains. The mechanisms in question span serotonergic, adenosinergic, and trophic factor-dependent pathways. Translational evidence highlights the potential of AIH to improve motor, respiratory, and cardiovascular function post-SCI with a favorable safety profile (139–141, 143–145).

### 6.3 Heart rate variability biofeedback

As previously described HRV analysis provides insight into autonomic regulation and has been used to monitor the severity and progression of autonomic dysfunction in various neurological conditions, including SCI (147–151). HRV biofeedback, which typically involves paced breathing at an individual's resonance frequency with real-time HRV monitoring, has shown promise in improving autonomic balance, enhancing baroreflex sensitivity, and reducing symptoms of anxiety and depression in chronic disease populations (152, 153). In SCI, a randomized controlled trial protocol is underway to assess whether a 10-week, once-weekly, computer-based HRV biofeedback intervention can improve autonomic and neural function, with outcomes including HRV, BP, pain, fatigue, and psychological status (136). While direct evidence in SCI is still emerging, HRV-biofeedback has demonstrated improvements in anxiety, pain, and motor performance in related neurological populations, such as post-stroke and mild traumatic brain injury patients, with protocols often involving weekly sessions over several months in both clinical and home-based settings (119, 152, 154). Systematic reviews suggest that HRV biofeedback is feasible, safe, and effective for autonomic self-regulation, with benefits observed after as few as six to ten sessions, though longer and more frequent training may yield greater improvements (152, 153). Home-based HRV biofeedback, especially when supported by digital tools, is increasingly viable and may enhance adherence and accessibility (153). Overall, HRV appears to be a promising biomarker of autonomic modulation in individuals with SCI and HRV biofeedback holds potential for improving autonomic regulation and associated symptoms; however, the current evidence remains heterogeneous and further high-quality studies are needed to more clearly establish its robustness and clinical applicability (148, 152, 153, 155).

### 6.4 Exercise and sympato-vagal balance

Alongside these biofeedback approaches, structured exercise programs are a well-established, yet underutilized, method of enhancing autonomic balance and cardiovascular resilience through activity-induced neuroplasticity (156–159). Recent research has focused on how different exercise modalities—particularly Moderate-Intensity Continuous Training (MICT) versus High-Intensity Interval Training (HIIT)—affect autonomic outcomes such as baroreflex sensitivity, catecholamine levels, and HRV in individuals with SCI (160–164). Exercise intensity is a pivotal, yet still incompletely understood, determinant of sympatho-vagal balance after SCI. Six-month programs of moderate-to-vigorous Arm-Cycle ErgomeTry (ACET) have been shown to consistently improve cardiovagal baroreflex sensitivity and HF-HRV, indicating a net parasympathetic enhancement without exacerbating orthostatic hypotension (99, 160, 161). However, while these improvements in outcome are reproducible, the underlying intensity-specific mechanisms remain unclear, as no head-to-head trials have directly compared MICT and HIIT using validated autonomic markers (162–165). HIIT may enhance sympathetic-somatomotor coupling and the release of neurotrophic factors (e.g., BDNF), thereby supporting neuroplasticity and improving autonomic regulation (22, 157, 159, 166). However, the benefits of higher exercise intensity must be weighed against an increased risk of AD, musculoskeletal overuse, and secondary tissue injury. This requires careful, device-assisted monitoring (167, 168). Current guidelines therefore strike a pragmatic compromise: at least 30 minutes of moderate aerobic exercise on five days per week, or at least 20 min of vigorous exercise on three days per week. This should be tailored to the individual's orthostatic tolerance, the completeness and level of the lesion, and any co-morbid cardiometabolic risk factors (156, 168). The choice of modality also affects autonomic outcomes. Upper-limb ergometry remains the clinical workhorse for cardiorespiratory conditioning in cases of tetraplegia and high paraplegia. However, its reliance on a small muscle mass limits sympathetic drive and may reduce catecholamine spill-over (169). In contrast, lower-limb Functional Electrical Stimulation (FES) cycling recruits' extensive afferent inflow from electrically driven muscles. This activates dormant spinal sympathetic circuits and amplifies cortico-spinal excitability. This process increases both cardiovascular reflex capacity and neuroplastic potential (157, 159, 170). However, direct microneurography at matched metabolic loads has not been performed, leaving questions about true sympathetic firing patterns unanswered (171). Similarly, although serum and CerebroSpinal Fluid (CSF) BDNF levels increase with training, the dose–response relationship between weekly functional electrical-stimulation-cycling volume and central neurotrophin dynamics remains unclear (156, 158). Hybrid modalities (e.g., functional electrical-stimulation-cycling combined with voluntary upper-limb exercise) show promise in maximizing autonomic and neuroplastic benefits, but evidence is still emerging (22, 159, 172, 173).

For individuals with high-level or motor-complete SCI, participation in high-intensity voluntary paradigms may be physiologically constrained by reduced active muscle mass, impaired sympathetic cardiovascular drive, and heightened susceptibility to orthostatic instability or AD. In this context, passive exercise modalities represent a clinically feasible and translationally relevant alternative. Passive Hindlimb Cycling (PHLC) has emerged as a promising rehabilitation strategy, particularly for individuals with high thoracic or cervical injuries who cannot generate sufficient voluntary motor output. By delivering rhythmic afferent input and cardiovascular stimulation without requiring active contraction, PHLC provides autonomic and neuroplastic engagement while minimizing metabolic stress (174, 175). Preclinical studies in complete high-thoracic SCI models demonstrate that long-term PHLC (4–10 weeks) reduces AD severity, decreases spontaneous hypertensive events, improves cardiac electrical conduction stability, and lowers arrhythmia susceptibility during sympathetic challenges (176–179). Early or delayed initiation of PHLC can prevent left ventricular atrophy and adverse cardiac remodeling, although these benefits diminish with detraining (174, 177). Mechanistically, PHLC appears to restore sympatho-vagal balance by attenuating unopposed parasympathetic dominance and normalizing aberrant spinal afferent sprouting (176). In contrast, shorter-duration protocols in incomplete injury models have shown limited effects on AD severity, highlighting the importance of injury completeness, training dose, and timing (180). Human data are more heterogeneous but increasingly supportive. Systematic reviews report improvements in peripheral circulation, reflex modulation, and muscle trophism, though effects on spasticity and long-term cardiovascular disease prevention remain inconsistent (175, 181). Acute passive leg cycling in individuals with tetraplegia modestly increases stroke volume, cardiac output, and endothelial function, but may transiently provoke AD, necessitating careful hemodynamic monitoring (182). Notably, an exploratory randomized clinical trial comparing passive treadmill training and active arm cycling found that passive modalities did not significantly improve orthostatic BP regulation, whereas active exercise enhanced cardiovagal baroreflex sensitivity, underscoring modality-specific autonomic effects (161). Collectively, these findings position passive cycling as a biologically plausible and translationally relevant strategy for early autonomic rehabilitation across a broad spectrum of SCI severities. While not yet standard-of-care for cardiovascular prevention, PHLC expands the therapeutic landscape for individuals unable to participate in high-intensity voluntary paradigms and may serve as a bridge toward more active or hybrid exercise modalities.

While these modality-specific adaptations underscore the therapeutic potential of exercise across injury severities, quantifying their autonomic impact remains challenging. Although HRV remains a promising biomarker of autonomic modulation in SCI, the current evidence on the effects of exercise training on HRV is inconclusive, particularly for high-level injuries (14, 99, 183, 184). Further research in this area is needed in order to reach definitive conclusions. Complementary markers, such as cardiovagal baroreflex sensitivity, 24-h Ambulatory Blood Pressure Monitoring (ABPM), and direct catecholamine measurements, may provide additional insights into autonomic regulation (14, 161, 183–185). Sympathetic Skin Response (SSR) and orthostatic challenge tests can also help to assess residual autonomic function and the risk of orthostatic hypotension (14, 163, 186). Overall, the evidence suggests that regular, individually dosed aerobic exercise, preferably combining voluntary upper-limb and electrically assisted lower-limb activities, should be prescribed to optimize autonomic balance and neural recovery in SCI. Based on the available evidence, several mechanistic gaps remain insufficiently addressed: (i) intensity-specific changes in autonomic markers and catecholamine kinetics, (ii) the training threshold at which the benefit–risk equation tips toward orthostatic instability, and (iii) the longitudinal, volume-dependent effects on neurotrophin-mediated plasticity. Rigorously controlled, multimodal trials addressing these questions will be essential for refining precision exercise prescriptions that maximize neuro-cardiovascular benefits while minimizing autonomic complications in this highly heterogeneous population.

### 6.5 Pharmacological treatment

The primary objective of drug treatment for SCI is to manage the consequences of ANS dysfunction in the acute and chronic phases. During the acute phase, an intact PNS, unopposed by SNS, reduces heart rate and BP, potentially causing cardiac arrhythmias. Treatment for patients with high cervical lesions may include atropine, vasopressors, or cardiac pacemakers. Guidelines for acute management have been defined by the (187). Vasopressors with alpha- and beta-adrenergic actions (including dopamine, NE and epinephrine) are typically the most effective option as they support the heart's rhythm while compensating for lost sympathetic tone. In the chronic phase, pharmacological treatment is used to prevent acute AD episodes after non-pharmacological measures have been exhausted. Anti-hypertensive medications are the most common form of management for acute and recurrent AD. Many pharmacological agents (i.e., nifedipine, nitrates, etc.) have been suggested for treating AD in a similar way to how hypertensive crises are treated in able-bodied populations. Among pharmacological agents that affect autonomic pathways, alpha-adrenergic blockers (such as prazosin) have been shown to significantly reduce the severity and duration of AD episodes in clinical and preclinical studies (9). Systematic reviews and clinical guidelines do not recommend using anticholinergic agents to regulate ANS in people with spinal cord injuries (9). Research involving patients with spinal cord injuries undergoing urodynamic testing found that, unless complete detrusor areflexia was induced, anticholinergic drugs did not prevent the onset of AD during bladder filling or distension (188, 189).

### 6.6 Personalized rehabilitation frameworks

SCI level and completeness clearly influence autonomic dysfunction, highlighting the need for an individualized approach. Higher-level injuries (cervical and upper thoracic) cause bradycardia, hypotension, orthostatic intolerance and AD, while the relationship between injury completeness and autonomic outcomes is less clear (184). Structured tools such as the International Standards to document Autonomic Function following SCI and 24-h ABPM allow standardized evaluation of cardiovascular instability and related phenomena (190, 191). Among these, HRV provides a sensitive, non-invasive biomarker for monitoring autonomic adaptations during rehabilitation and tracking responses to therapeutic interventions, although its indices must be interpreted cautiously in SCI (102, 184). Despite the availability of these tools, no comprehensive algorithm currently integrates autonomic, clinical, neurophysiological, and functional data to guide rehabilitation strategies in SCI (184). Recent advances in robotics Virtual Reality (VR), neuromodulation, and AI-driven multimodal modeling are rapidly advancing neurorehabilitation toward adaptive, individualized paradigms (16, 192–195). Nevertheless, the integration of autonomic markers within these frameworks remains largely experimental, emphasizing both the originality and urgency of developing validated, SCI-specific algorithmic approaches. The incorporation of wearable biosensors and regular autonomic testing may further enhance real-time monitoring and adaptive therapy management, although robust clinical validation is still needed (183, 184). Overall, although methodological foundations for biomarker-driven personalized rehabilitation are emerging, the establishment and validation of evidence-based algorithmic workflows remain critical priorities to optimize both autonomic and neuromotor recovery in individuals with SCI.

## 7 Limitations

The present scoping-like review is subject to certain inherent limitations. Notably, the absence of systematic inclusion criteria and the lack of formal quality assessment may introduce selection bias in the sources consulted (196). While this approach enables a flexible synthesis of emerging evidence across diverse domains, it inherently limits the replicability and generalizability of findings compared to systematic reviews or meta-analyses. Moreover, a substantial portion of the reviewed literature is derived from studies employing heterogeneous methodologies and involving diverse clinical populations. This variability may affect the consistency and robustness of the conclusions drawn, underscoring the need for future research employing standardized protocols and well-defined outcome measures.

## 8 Conclusion

Autonomic modulation has been identified as a pivotal mechanistic factor in the context of neuroplasticity and functional recovery following SCI. Contrary to the prevailing view of autonomic modulation as a secondary consequence of injury, recent research has demonstrated its role as a central mechanistic determinant. Recurrent sympathetic surges, as exemplified by autonomic dysreflexia, have been demonstrated to bias spinal and supraspinal networks toward maladaptive remodeling. This process has been shown to suppress neurotrophin signaling, promote microglia-mediated synaptic pruning, and compromise vascular and metabolic support for plasticity.

Conversely, enhanced vagal engagement and parasympathetic flexibility have been shown to restore homeostatic balance, increase the availability of BDNF–TrkB and NGF–Trk trophic pathways, stabilize neuroimmune interactions, and support adaptive circuit remodeling. Interventions such as VNS, AIH, HRV biofeedback, and structured aerobic exercise have demonstrated the potential to harness autonomic tone therapeutically, amplifying activity-dependent plasticity in spared motor, respiratory, and autonomic pathways. These findings suggest that autonomic-targeted strategies could be a promising axis for precision-based rehabilitation. These strategies would be capable of integrating neurophysiological, vascular, and immunomodulatory mechanisms to optimize recovery.

Future research should pursue rigorously powered, multimodal clinical trials that incorporate autonomic biomarkers, particularly HRV and blood pressure variability, alongside neurophysiological endpoints. Such an approach will facilitate the establishment of dose-specific, individualized protocols, thereby integrating mechanistic insights with clinical translation and, in turn, propelling the advancement of personalized rehabilitation paradigms for SCI.
